# A messy reality: an analysis of New Zealand's elective surgery scoring system via media sources, 200–2006

**DOI:** 10.1002/hpm.2127

**Published:** 2012-07-20

**Authors:** Sarah Derrett, Kim Cousins, Robin Gauld

**Affiliations:** 1 Commonwealth Fund Harkness Fellow in Health Care Policy and Practice; & Injury Prevention Research Unit, Department of Preventive and Social Medicine, Dunedin School of Medicine, University of OtagoDunedin, New Zealand; 2 Department of Preventive and Social Medicine, Dunedin School of Medicine, University of OtagoDunedin, New Zealand

**Keywords:** waiting list, elective surgery, prioritisation, rationing, New Zealand

## Abstract

Waiting lists for elective procedures are a characteristic feature of tax-funded universal health systems. New Zealand has gained a reputation for its ‘booking system’ for waiting list management, introduced in the early-1990s. The New Zealand system uses criteria to ‘score’ and then ‘book’ qualifying patients for surgery. This article aims to (i) describe key issues focused on by the media, (ii) identify local strategies and (iii) present evidence of variation. Newspaper sources were searched (2000–2006). A total of 1199 booking system stories were identified. Findings demonstrate, from a national system perspective, the extraordinarily difficult nature of maintaining overall control and coordination. Equity and national consistency are affected when hospitals respond to local pressure by reducing access to elective treatment. Findings suggest that central government probably needs to be closely involved in local-level management and policy adjustments; that through the study period, the New Zealand system appears to have been largely out of the control of government; and that governments elsewhere may need to be cautious when considering developing similar systems. Developing and implementing scoring and booking systems may always be a ‘messy reality’ with unintended consequences and throwing regional differences in service management and access into stark relief. Copyright © 2012 John Wiley & Sons, Ltd.

## BACKGROUND

Waiting lists are a characteristic feature of tax-funded universal health systems (Siciliani and Hurst, 2005; Willcox *et al*., 2007). The basic assumption is that the demand for specialist elective treatments simply outweighs the capacity to provide (Rotstein and Alter, 2006). Over the past decade or so, waiting lists have received considerable research attention with studies highlighting contributing factors. These range from the under-supply of health human resources and inadequate funding to the need to reorganise the systems for managing elective services and patient flows (Ham and Robert, 2003; MacCormick *et al*., 2003; Siciliani and Hurst, 2005; Willcox *et al*., 2007). A key theme has been that, with better or more systematic methods of organisation, waiting lists will reduce. Furthermore, the means for prioritising elective patients must be transparent and fair so that each patient is treated equally and in order of need and has some certainty about where they are on the list and when they are likely to receive treatment. A long-standing argument is that waiting list management—who gets treated first, and how long they should wait—has been subject to professional judgement and, therefore, open to abuse (Hadorn and Holmes, 1997). Research has also considered the impact of waiting on patients, with findings that those awaiting necessary treatments often face considerable costs. These may be financial if the ability to work is affected and if there is a need to pay for additional care and therapeutics while awaiting treatment. Costs for the health system may arise if patients are not treated in a timely manner and develop more serious conditions or co-morbidities as a consequence of waiting. There may also be quality-of-life impacts, as well as impacts on family or caregivers (Derrett *et al*., 1999; Harrison and Appleby, 2009; Kreindler, 2010).

Governments have pursued various solutions to the waiting list problem. Some, such as Britain and Denmark, have produced targeted times within which treatment should be provided for patients deemed eligible (Strandberg-Larsen *et al*., 2007; Harrison and Appleby, 2009). Others, such as Canada, have sought to make transparent the numbers of patients on different hospital waiting lists and numbers taking longer than a specified time to be treated (Willcox *et al*., 2007). Such approaches are designed to focus providers on providing timely service. A ubiquitous response has been increased funding for elective services, combined with attempts to streamline waiting list management and the administration of health professionals and facilities such as operating theatres. Governments have also contracted private practitioners to provide services for publicly funded patients.

New Zealand has gained a reputation for its ‘booking system’ for waiting list management, development of which commenced in the early-1990s. New Zealand's health system is 80% government funded from general taxes. Public hospitals are dominant, universally accessible and free of patient charges; private hospitals provide only elective services to full-fee paying patients (Gauld, 2009). Naturally, there are waiting lists for elective services. The New Zealand booking system features two components. The first is standard criteria that health professionals use to score various aspects of a patient's condition so that patients with higher scores and, in theory, greater need are able to be given priority access to treatment. In other words, they are placed higher on the waiting list than people with lower scores. Those with scores below a certain threshold—ideally, that clinicians deem to be the point that surgery or other specialist treatment is not required—are not placed on waiting lists and, instead, directed back to their referring primary care provider. The second component is the booking of patients—as with an air travel booking—aimed at providing certainty for patients in that they will be treated at a specific time and date (Gauld and Derrett, 2000).

The New Zealand booking system has been widely studied and discussed, with advocates suggesting that it provides a tailor-made solution to the problems stemming from traditional waiting lists (Hadorn and Holmes, 1997). The government intended the booking system to address many identified problems with the former queue-style waiting list. The booking system was to be, for example, a fairer and consistent means for determining access to treatment, to allow for transparent decision making, to provide certainty to patients about the timing of treatment and to provide nationally consistent access to surgery (National Advisory Committee on Core Health and Disability Support Services, 1995; Shipley, 1996; Ministry of Health, 2000). However, studies have also demonstrated that the system, although great in theory, has been troubled in practice (Gauld and Derrett, 2000; McLeod *et al*., 2004b). Problems encountered in the early years of implementation (1993–2000) have been outlined in prior articles by the authors and others (Seddon *et al*., 1999; Gauld and Derrett, 2000; Roake, 2003; Derrett *et al*., 2009). Despite substantial government and service provider attempts to address the difficulties, the system remains far from ideal. Considerable regional differences in access to elective services persist; there remain shortfalls in funding and capacity to treat all booked patients, meaning many are returned to referring primary care providers for ‘active review’; and the 20 District Health Boards (DHBs),^1^ that have devolved responsibility for planning and providing publicly funded services for their respective geographic regions, tend to each have different approaches to managing elective services demand. This is despite a supposed national policy framework.

Healthcare has consistently been a top-ranked concern of the New Zealand public in opinion polls, and the New Zealand media pay close attention to events in the health system. Following this, waiting lists and the booking system are never far from the media's gaze, and any local DHB policy change or story about impact of national policy on local services tends to reach the local newspaper headlines. In this article, we discuss such issues by providing an analysis of the scoring and booking system through the period 2000–2006. We do so through the lens of the media, drawing on articles in daily newspapers, supplemented by Parliamentary Questions. Despite having a unitary political system, with a central national government, New Zealand has never had a national daily newspaper. So, for both New Zealand residents and those from abroad, it is very difficult to gain a clear picture of the national situation with the booking system. There is also no national report, produced by government or any other entity, comparing booking system policies and developments in different regions. This said, there is a Web-based data set that provides comparisons across DHBs of numbers of patients waiting for first specialist assessment, of numbers of patients booked for treatment and, following booking, of numbers waiting longer than 6 months for treatment (the government's target within which treatment should be provided). However, this is simply a descriptive set that historically has no information about local contextual issues and DHB policies that affect the shape and function of the booking system.

The aims of this article are to (i) provide a ‘regional’ perspective on the booking system, describing the key issues focused on by the media; (ii) identify local DHB strategies proposed, or implemented, to address identified difficulties; and (iii) present evidence of regional variation via newspaper media reporting on the booking system.

## METHODS

Newspaper sources were searched from 1 January 2000 to 31 December 2006, inclusive. This period was chosen for several reasons. The booking system was officially introduced by the then Minister of Health, with much media and public interest, in 1996. There was then a period of some years during which the hospital groups (DHBs from 2000) and booking system management processes were being established and modified. This article is focused upon the system ‘as established’—not the system during its ‘time of introduction’—hence, our focus on media reports from 2000 onwards. The closure date for the analysis was pragmatically selected to mark the first decade since the system was introduced.

While there is no national daily newspaper, all but one of New Zealand's regionally based newspapers are owned by a single parent company, Fairfax Media Limited, which also has considerable news media ownership in Australia. Stories from Fairfax New Zealand newspapers are indexed in a national news database, Newstext, that provides full-text archival access. Additional news stories are indexed in Factiva, another database that also includes non-Fairfax sources.

We used both Factiva and Newstext to search stories from all major print sources. The Factiva database includes all articles from *The Dominion/Dominion Post, National Business Review, New Zealand Herald, New Zealand Press Association, Otago Daily Times, Sunday Star Times* and *The Independent Financial Review* for the period 2000–2006. The Newstext database includes *Sunday Star Times, The Dominion/Dominion Post, New Zealand Herald, Waikato Times, Sunday News, The Evening Post, The Press, The Timaru Herald, The Evening Standard, The Daily News, Truth, The Southland Times, Taupo Times, Nelson Mail, Whangarei Leader, Stuff* and *Scoop Independent News*. Stories from the first three newspapers were excluded from the Newstext search, as they were already within the Factiva search. Although articles posted on *Stuff*, an online news website, and as *Radio New Zealand* Newswires, were also available from Newstext, these were only included if they had not already been published in other sources.

On each sitting day, local Members of New Zealand's Parliament are able to ask questions of government ministers around issues that affect their local constituents such as the hospital booking system. Therefore, to supplement the news media stories, we also drew on information that related to the local implementation of the booking system elicited through New Zealand's Parliamentary Question Time. Text of the resulting exchange is also available electronically via Newstext and was searched in the same way as for the newspapers. In keeping with the aims of our study, we excluded Parliamentary question information related to national policy around the booking system.

### Data collection

Focusing on 2000–2006, we first performed a simple keyword search in both Factiva and Newstext to identify all relevant stories. Keywords could be anywhere in the full article (not just the headline). The following keywords were used: elective surgery (Factiva: 1006 hits; Newstext: 1374 hits), booking system (Factiva: 267 hits; Newstext: 519 hits), waiting list (Factiva: 2255 hits; Newstext: 6392 hits), non-urgent surgery (Factiva: 226 hits; Newstext: 184 hits) and active review (Factiva: 128 hits; Newstext: 202 hits).

Each resulting news item was manually checked by at least two members of the research team to ensure that it met our criterion for analysis, which was, very broadly, anything to do with the elective surgery booking system. Stories therefore included a range of issues such as coverage of local responses to national policy, hospital announcements, patients affected by waiting or changes in access criteria as a result of funding shortfalls or changes, health professionals' views of the system, and government policy announcements and commentaries.

When duplicate entries were removed, a total of 1199 stories relating to the elective surgery booking system had been identified. These were analysed and grouped into key categories and in chronological order through the period under study. Categories were then reviewed to identify thematically the types of issues or strategies being covered in each article. In many cases, several newspapers ran similar stories within a day or two of one another on a new development in, or local response to, national booking system policy. Each would tend to provide local contextual information such as local impact of the policy, data about numbers of patients not receiving timely treatment and material from interviews with local surgeons, managers or patients.

**Figure 1:**
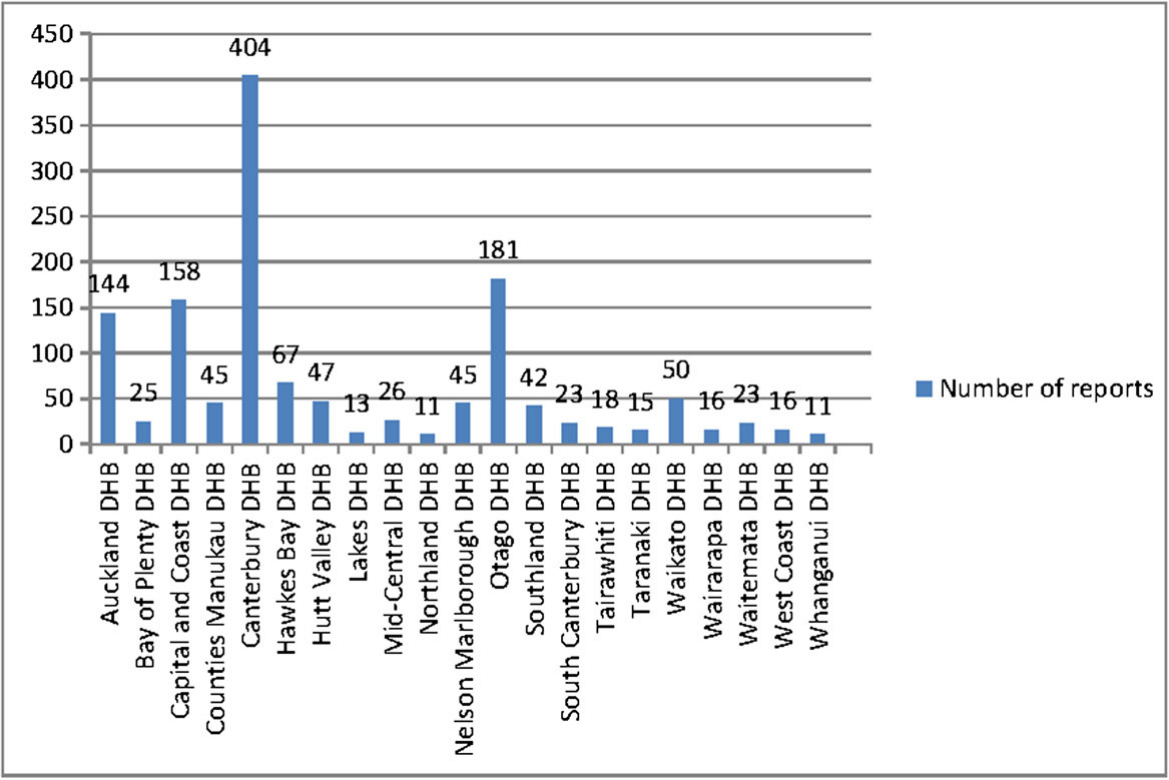
Number of media reports (2000–2006) according to District Health Board

After preliminary analysis, the 1199 media reports were grouped into categories as follows: location (region the media report was covering, as per Figure 1), opinion (whose opinion was being reported), health service/group affected, type of media article (e.g. editorial or personal story), disruption (stories describing disruption to booking system), explanation (explanations offered for booking system disruptions), strategy (strategies for improving the booking system), proposed solutions (to address problems), policy initiatives covered and outcomes described. Media reports were then coded into themes within each category by using an iterative process in which team meetings were held to develop a list of possible codes, and then new codes were added by agreement. All reports were coded to at least one theme, but often more than one. For example, a story about a decision to ‘cull’ the waiting list would be simultaneously coded to the DHB region affected by the cull, the patients affected by the cull, the health professionals expressing an opinion about the decision and the policy initiatives reported to be the cause of the cull. Two people coded the stories, and one person verified coding for all stories at the end to ensure all were coded to ‘new’ codes that emerged during the analysis.

## FINDINGS

Some DHBs were more affected by waiting list difficulties than others, as reflected in the volume of newspaper stories (Figure 1). The Christchurch Press carried by far the highest number of reports on its DHB (among the largest), which routinely faced the range of challenges discussed in the succeeding sections. Key issues that cut across the categories and themes (Table 1) that emerged through the 7-year period of analysis are further discussed in the sections that follow. These include the following:

- Staff shortages in certain medical specialties and New Zealand's capacity to offer internationally competitive salaries undermined the capacity to provide timely or adequate volumes of service.
- Government periodically announced new funding injections following high-profile incidents of waiting list growth, or incapacity to provide services, and evidence that this negatively affected groups of patients and, in turn, perceptions of government performance.
- Government periodically announced new policy initiatives aimed at more efficient waiting list and booking system management.
- DHBs routinely removed scored and eligible patients from waiting lists because of incapacity to treat them, blaming differing factors for this. This was despite many patients receiving scores above the points threshold that deemed them to be in clinical need of treatment.
- Acute and emergency services influenced capacity to perform elective procedures.

**Table 1:** Newspaper articles (*n* = 1199) by category and theme

Category	Articles	
	
Theme	*n*	%
Affected group/service		
General surgery	486	40.5
ENT	407	33.9
Orthopaedic surgery	188	15.7
Heart patients	173	14.4
GP managed care/referrals and primary care	108	9.0
First specialist appointments	88	7.3
Eye patients	78	6.5
Explanation		
Staff shortage	232	19.3
List cull	201	16.8
List management	195	16.3
Lack of funding	132	11.0
Threshold	112	9.3
Service disruption		
Strike action	151	12.6
Acute overload	80	6.7
Bed shortage	28	2.3

GP, general practitioner; ENT, ear, nose and throat.

### Human resources

A persistent theme in each of the years from 2000 to 2006 was to attribute hospital inability to provide timely access to elective services to staff shortages. ‘Christchurch patients are having their planned operations pulled and others are not being booked, owing to a national shortage of anaesthetists’, reported the Christchurch Press in 2001 (Martin, 2001), whereas the *New Zealand Herald* later wrote that

‘Hundreds of children are being forced to wait longer for surgery at the country's leading paediatric hospital as it struggles with a shortage of theatre nurses. Many patients at the Starship children's hospital in Auckland already wait far longer than the Government benchmark of six months for non-urgent surgery, and the delays have got worse’ (Johnson, 2005a).

Shortages in clinical specialties such as anaesthetics, ophthalmology and urology, as well as in nursing, all contributed. These shortages were reported to impact on waiting lists and the booking system in a number of ways. Inadequate staffing meant there was a frequent shortfall in the capacity to provide the volume of service required to treat all people who received enough points to be eligible for treatment. As a result, the number of patients waiting expanded, bringing into question whether the target of treatment within 6 months could be met. The implication was that patients with lower priority scores were at risk of not being treated in a timely manner. With recruitment the sole responsibility of local DHBs, staffing shortages affected some regions more than others, which was a strong theme in media stories. Thus, waiting lists and equitable access to elective services were also affected by regional capacity to maintain a full and stable workforce.

### New funding

In all but one of the 7 years under study, the government announced new, additional funding for elective services with the following quote from the Christchurch Press typical of reporting on the issue:

‘The Government is injecting an extra $200 million into non-urgent surgery—enough money for about 10,000 more operations a year. Yesterday's funding announcement by Health Minister Pete Hodgson comes amid growing public unease about the Government's health policies. Hodgson said the four-year package would be available to all health boards from next month, as long as they complied with its booking policy. The move comes after months of pressure from patients, doctors and politicians as thousands of patients were dropped from waiting lists’ (Davis, 2006).

The new funds were mostly *ad hoc* introductions and followed considerable political and practical pressure for action. For example, in 2000, extra funding was provided initially for specific specialties (orthopaedics, (ENT) ear, nose, throat, and cardiac surgery) that had substantial waiting times and lists. This was followed by a significant injection, announced in the 2000 Budget, for each of the next 4 years (Boland, 2000). Despite this, many DHBs remained in deficit (meaning that they provided more services to meet demand than they received government funding for), which they are required by the government to trade out of, making it difficult for them to build the capacity to provide the services financed by elective services funding injections. In November 2003, at least two DHBs announced elective service cutbacks in the effort to reduce budget deficits (New Zealand Press Association, 2003a, 2003b). In 2002, new funds were provided for cardiac services in the South Island of New Zealand and, in 2003, for cardiac services in the lower North Island (Staff Reporter, 2003). Growing pressure on orthopaedic waiting lists led to the 2004 ‘orthopaedic initiative’, which provided specific funding for the subsequent 4 years tied to increasing access to orthopaedic services. Again, DHBs noted limited capacity to deliver new operations within a restrictive general financial environment (Mayston, 2005; Walsh, 2005).

### New funding = improved access?

There has been an ongoing political debate in New Zealand about whether funding injections result in increased and improved service access. Between 2000 and 2006, there was a slight reduction in the total number of people receiving elective treatments (Ministry of Health, 2008). However, the Health Minister suggested that the reduction was a result of more treatments in outpatient settings that were not captured in standard hospital datasets. A clear theme throughout the 7 years of analysis was the constant stories of DHBs having difficulty providing adequate service levels and of patients being removed from waiting lists despite the fact that they had a professionally determined need, as judged by their clinical priority scores, for treatment (New Zealand Press Association, 2003a; Johnson, 2006a). Another theme was the increasing threshold, or required score, for access to elective treatments (Nichols, 2003b; Welham, 2003; Johnson, 2004; McCracken, 2004). Many DHBs, under pressure to provide a response to growing numbers of referred patients, simply raised the number of points required to be eligible for treatment (Parliamentary Question Time, 2002; Ross, 2003). As the *New Zealand Herald* reported:

‘The figures show the mean score for adults having cardiothoracic surgery has risen from 33.5 in 2001 to 46.4 in 2005. Mean adult general surgery scores have risen from 77.5 to 87.9 and orthopaedic scores from 75.4 to 81.2 over the same period. There has also been a big jump in ophthalmology scores’ (Berry, 2006).

So, although funding may have been aimed at improving access, access became increasingly contingent on being worse off in the first place (MacDonald, 2006).

### New policy initiatives

Between 2000 and 2006, there were three major new national policy initiatives designed to improve elective services access and booking system management. First, the ‘active review’ policy was introduced in 2000. This was aimed at providing ‘certainty’ for the large number patients then waiting longer than 6 months for treatment. However, there remained, at end-2006, a considerable number of patients subject to active review plans (Mayston, 2005). This policy required that DHBs develop a plan for monitoring and supporting patients in community settings. A plan may include a general practitioner visit and prescribe drugs. Second, the aforementioned ‘orthopaedic initiative’ that was aimed at improving access in an area subject to ongoing troubles, and with evidence of increasing demand from an aging New Zealand population requiring more hip and knee joint operations (Hayman, 2006b). Third, the linking of new funding, announced in the 2006 Budget, to concrete evidence that funded treatments can be provided (Katterns, 2006). This meant that DHBs had to show they had the necessary physical and human resources before they would be eligible for new elective money, and stemmed from an unpublished Ministry of Health study of how to improve the performance of the booking system and elective services. Media reports suggested that ‘Under its much-vaunted orthopaedic initiative, the Government promised to double the number of major joint replacements by 2008. But DHBs have also been told to only offer surgery to patients they can treat within six months’ (Hayman, 2006b).

### Patient culling

A key method for elective services management was to remove from waiting lists patients who had previously been scored and informed that they had been eligible for treatment. This occurred as DHBs had simply been unable to foresee a situation where they would be able to provide all eligible patients with treatment within government-targeted timeframes (Watkins, 2006). In other words, government funding was insufficient, regardless of the fact that the scoring system brought transparency to the process of determining the actual need for service in the community. As well as new funding, the government and DHBs searched for ways to align the capacity to provide elective services with demand. In 2001, a significant number of patients (over 50%) were effectively removed from the list of those booked for surgery under the ‘active review’ policy, noted earlier. As one newspaper reported,

‘Six thousand more patients have been dropped from surgery waiting lists, with no prospect of treatment under existing funding limits, Health Ministry figures issued yesterday show. The increase in the number of patients in the “active review” category—where they will receive treatment only if they deteriorate enough or more funding becomes available—takes the total to 24,400. The ministry's quarterly waiting report says it is critical that these patients are monitored and their plan of care and treatment status updated, but notes concern that some DHBs do not have processes in place to manage these patients’ (Birnie, 2001).

The removals were essentially because many had been waiting longer than the targeted 6 months for treatment and therefore were considered low priority. The policy had the greatest impact on DHBs with larger lists of such patients and continued to be applied every year after 2001. Removal decisions were taken by booking system managers, without involvement of clinical specialists. Managers suggested that active review (essentially confirming that affected patients will not be re-booked for treatment until their conditions significantly worsen) was about ‘being transparent about [patients'] hopes and being quite clear what the expectations are for them’ (Nichols, 2003a).

In 2006, the government introduced new financial penalties for hospitals that failed to provide treatment for booked patients within 6 months. This induced a new round of culling, affecting patients from at least one-third of DHBs. Over 8000 patients were removed from lists. In a highly controversial case, one of the largest DHBs removed a few thousand patients and, following considerable public pressure, then found money to reinstate a small number (around 200) whom specialists deemed in urgent need of treatment (New Zealand Press Association, 2006). By this time, some had already paid for private surgery (Hayman, 2006a).

### National inconsistency

As mentioned, since the introduction of the booking system in the mid-1990s, it has been government policy that there should be nationally consistent access to elective services in New Zealand's taxpayer-funded, universally accessible health system. In the early years of the booking system, different scoring tools were used in each hospital, meaning it was impossible to compare whether access was equitable (Gauld and Derrett, 2000). Since 2000, there has been a concerted effort to develop national consistency, and by end-2006, the government reported that around 97% of patients were scored using comparable tools (Ministry of Health, 2007). However, through the study period, access inequities remained a persistent theme with some DHBs providing more than double the number of elective treatments than others. Moreover, throughout 2000–2006, DHBs set their own levels of points required to be booked for treatment, and these levels tended to be managerially driven, in response to available funding, and bore no relationship to clinically determined need (Smith, 2004b; Johnson, 2006b; Ryall, 2006). One news report featured a DHB manager noting that ‘In Otago you need to have a higher number of points than elsewhere in order to be eligible for treatment…’ (Smith, 2004b). This meant that access to treatment was dependent upon place of residence. Reflecting this, another newspaper reported that:

‘Access to cataract surgery, rationed on national criteria, is uneven around the country. Some boards give it to people who score 21 out of a maximum 100 points for things such as degree of visual impairment and ability to work. Others exclude much worse-off patients who have more than 30 points’ (Johnson, 2005b).

### Acute and emergency services

A final theme, consistent over the 7 years, was capacity to plan and manage elective services, which was affected by the demand for acute and emergency services. As reported in the *Dominion Post*, ‘…in the main centres, unexpectedly high numbers of urgent patients pushed out waiting list patients’ (MacDonald, 2003). Public hospitals provide most of New Zealand's publicly funded elective treatments alongside all acute and emergency care: no private New Zealand hospital offers urgent services. In each year of this study, DHBs noted that acute and emergency services take priority over electives, and when demand for the former surges, planned electives are postponed. Episodically, hospitals cancelled elective treatments for a period to cope with surging urgent service demand (Smith, 2004a; Kiong, 2006).

## DISCUSSION

Previous studies and reports have implied that the New Zealand scoring system provides a ready-made solution to waiting list management problems (Hadorn and Holmes, 1997). The argument is that scoring systems, if implemented, provide transparency to the process of clinical judgement over who receives specialist elective services and in what order; they also provide firm evidence of the actual need for elective services in a population. Yet, other studies have shown that the system is not without its troubles (Roake, 2003; McLeod *et al*., 2004a, 2004b; Dew *et al*., 2005; Derrett *et al*., 2009). These studies have found that clinicians may game the system to ensure that patients receive enough points for treatment; that there are problems with inter-rater reliability of points scoring systems; and that the development of scoring criteria is itself a subjective process, influenced by the preferences of clinicians. For instance, higher weightings, which impact on patients' overall scores, might be given to clinical issues with limited weighting on the impact of the condition on a patient's quality of life (Derrett *et al*., 1999). There are also multiple possibilities in terms of the types of scales and criteria that might be used to compose a scoring tool (MacCormick *et al*., 2003).

There have been no studies to date of the impact of contextual pressures on, and incremental changes to, local booking system policy and management. The research reported in this article was, therefore, designed to fill this gap and to highlight the policy, management and organisational factors that affect the capacity to develop and maintain a nationally consistent scoring system for elective services waiting list management in New Zealand. The findings—that issues such as human resourcing and the *ad hoc* introduction of different sorts of policies to improve waiting list management and reduce the numbers of patients falling outside targeted treatment times—illustrate that a wide range of factors are evident. Any one of these could have a bearing on national policy consistency and equity of access. Furthermore, they demonstrate, from a national system perspective, the extraordinarily difficult nature of maintaining overall control and coordination. As noted, an influx of emergency patients in one region or staff shortages in another can undermine a well-organised booking system, impacting on inter-regional equity of access. Equity and national consistency is also affected where an individual hospital responding to local pressure increases the number of points required to receive specialist elective treatment. Where all the factors outlined in the findings section work in tandem, as has often been the experience in New Zealand, this creates a particularly intractable set of circumstances.

There are three implications of the above. First, if national consistency of policy and access is a goal—and it ought to be in a national, tax-funded and universally accessible health system such as New Zealand's—then central government probably needs to be closely involved in local-level management and policy adjustments. As indicated in this article, for many New Zealanders, elective service access has been dependent on where they live. Following this, it may be inadvisable for a government to cede control over components of a national scoring system to local managers and planners. That said, as noted elsewhere, there is always going to be a tension between national control and local autonomy in line with the need to give managers and clinicians the capacity to respond to local needs (Saltman *et al*., 2007). The second, and perhaps more serious implication, is that through the study period, the New Zealand scoring system appears to have been largely beyond the control of central government, given the aim of national consistency and inability to achieve this. Despite ongoing efforts to build national consistency and equity into the system, which commenced in a concerted manner in 1998, the material presented in this article suggests that it remained as troubled at the close of the study period as it did a decade prior (Gauld and Derrett, 2000). Third, governments elsewhere may need to be cautious and look closely at experiences such as New Zealand's when considering developing elective services scoring systems. The New Zealand experience demonstrates that it is difficult for a central government to maintain a consistent approach with scoring and booking systems. This points to the conclusion that, in reality, developing and implementing scoring and booking systems may always be a ‘messy reality’ with unintended consequences such as highlighting variable use by and support for such systems amongst surgeons, and throwing regional differences in service management and access into stark relief.

The arguments in this article may be limited by the fact that they are largely derived from media sources. However, the sources include a large number of regional daily newspapers with reports on similar subjects produced by different journalists in different regions of New Zealand. Furthermore, we supplemented media reports with material from Parliamentary Question time where politicians are required by Parliamentary law to provide accurate and honest answers to questions. The advantage of our method is that it provided access to a range of local experiences and issues over a 7-year period in a way that could not be otherwise captured. In this respect, this article provides a key contribution to the understanding of scoring and booking systems in that it provides a bottom-up view of the implementation of national policy. As others have shown, this perspective can be useful for understanding factors that may be pivotal to policy success or failure (Exworthy *et al*., 2002; Hill and Hupe, 2002).

The core argument in this article is that, through a decade of considerable development and use, the booking system, at least as New Zealand experiences viewed through the lens of the newspaper media from 2000 to 2006 imply, was far from a panacea. Our analysis—a regional snapshot of the national implementation of the system—shows that its application was not systematic: that there had been periodic changes in central government policy, largely following high-profile negative incidents, as well as substantial capacity for regions to incrementally change the way in which they administer the system.

Although the research in this article covered the 7-year period of 2000–2006, there is reason to believe that the focus of elective services management has changed more recently. A government elected in 2008 made elective surgery one of six new health targets and developed a new process for public reporting. This involves publishing quarterly comparative performance scores for 20 DHBs as measured against a target of increasing the number of procedures performed (Ministry of Health, 2011). Thus, the focus has shifted from the process of managing patients as they move through the system of assessment to treatment, including clinical priority scoring, to one of results measured by the sheer number of procedures. However, a recent Auditor-General's report has revealed continuing difficulties with appropriate prioritisation, equitable access and national consistency (Controller and Auditor-General, 2011).

## Footnotes

1 There were 21 DHBs from their creation in 2000. A merger in 2010 reduced the number to 20.
